# Clinical Observation of Acupuncture Combined with Biofeedback Electrical Stimulation in the Treatment of Female Stress Urinary Incontinence

**DOI:** 10.1007/s10484-024-09653-2

**Published:** 2024-07-22

**Authors:** Na Tian, Jingsi Bai, Jie Li, Caiqing Ji

**Affiliations:** 1 Department of Gynecology, Hebei General Hospital, Shijiazhuang, 050000 China; 2 Department of Nursing, Hebei General Hospital, NO.348 of Heping west Street, Xinhua District, Shijiazhuang, 050000 China

**Keywords:** Stress urinary incontinence, Acupuncture, Biofeedback electrical stimulation

## Abstract

To investigate the clinical efficacy of acupuncture combined with biofeedback electrical stimulation on female stress urinary incontinence. Ninety patients diagnosed in a hospital between January 2020 and January 2021 were randomly divided into three groups (A, B and C). Group A was treated with biofeedback electrical stimulation, 3 times a week for 30 min for 15 times. Group B used acupuncture treatment, including *Guanyuan*, *Qihai*, *Zhongji*, *Zusanli*, *Sanyinjiao* and *Yinlingquan*, once a day, Monday–Friday, 30 min each, for a total of 10 times. Group C was treated with acupuncture combined with biofeedback electrical stimulation. All three groups were combined with pelvic floor muscle training. Following treatment, the changes in class I and II muscle fibre strength, ICI-Q-SF score and urine leakage in the 1-hour pad test were compared. Prior to treatment, there was no significant difference in the general data of the three patient groups, as well as class I and II muscle fibre strength, ICI-Q-SF score and 1-hour urinary pad test (*P* > 0.05). Following treatment, class I and II muscle fibre strength in groups A and C improved compared with before, with statistical significance (*P* < 0.05); there was no significant difference in group B (*P* > 0.05). In the three groups, ICI-Q-SF scores and 1-hour urinary pad test results were lower compared with before (*P* < 0.05), with those in group C better than those in groups A and B (*P* < 0.05). The treatment efficiency of the three patient groups was 86.7%, 83.3% and 96.7%, respectively. Combined acupuncture and biofeedback electrical stimulation can improve pelvic floor muscle strength, urine leakage and quality of life, and can be superior to biofeedback and acupuncture treatment alone.

## Introduction

Stress urinary incontinence (SUI) refers to urinary incontinence that occurs when abdominal pressure increases, such as when coughing, laughing, sneezing and during exercise (Billecocq et al., 2019). Foreign studies have shown that the incidence of SUI is 14.9% (Legendre et al., 2020). The prevalence of SUI among adult women in China is as high as 18.9% and is increasing year by year (Chinese Medical Association Obstetrics and Gynecology Branch Gynecology Pelvic Floor Group, 2017) seriously affecting the physical and mental health of women and attracting the attention of medical staff. At present, the most widely used non-surgical treatment methods are biofeedback electrical stimulation therapy and traditional Chinese medicine acupuncture therapy (Zhang et al., 2017). Biofeedback plays an important role in SUI therapy (Hagen et al., 2020; Liu et al., 2018). Biofeedback electrical stimulation makes the muscles passively contract by electrically stimulating the pelvic floor muscles or nerves, with the biofeedback guiding specific muscles to actively contract, which can effectively improve the patient’s pelvic floor muscle strength and any urinary leakage symptoms; however, the treatment time is long and the compliance is poor. The therapeutic effect of acupuncture on SUI has been confirmed (Shi et al., 2022; Yang et al., 2020). Acupuncture treatment can effectively improve the symptoms of urinary leakage by dredging the meridians, warming the kidneys and fixing the intake, and has a quick onset and short course of treatment. In this paper, acupuncture combined with the biofeedback electrical stimulation method is used to study the efficacy of SUI treatment and provide a reference for clinical treatment of SUI.

## Materials and Methods

### General Information

A total of 90 patients who were diagnosed with SUI in a gynaecological pelvic floor clinic of Hebei General Hospital between January 2020 and January 2021 were selected as the research participants.

The inclusion criteria were as follows: patients (1) who met the diagnostic criteria of SUI; (2) aged over 18 years old and with a history of sexual life; (3) undergoing no treatment related to SUI within 3 months; (4) with no mental illness and without cognitive dysfunction, able to cooperate with examination and treatment; and (5) patients voluntarily participating and signing the informed consent.

The exclusion criteria included: (1) combined with urge urinary incontinence, uterine prolapse, vaginal anterior and posterior wall prolapse; (2) pregnancy, postpartum lochia or abnormal vaginal bleeding; (3) acute phase of inflammation (pelvic cavity, vagina, urinary system); (4) patients with metal substances such as pacemakers and metal stents in the body; (5) denervation of pelvic floor muscles (no sensation, no contraction); and (6) coagulation dysfunction.

Using SPSS (ver. 26) software(IBM, Armonk, NY, USA), the patients were randomly divided into three groups (groups A, B and C) with 30 in each group. Medical records for the patients were established and their general information was collected, including age, height, weight, course of disease, number of deliveries, mode of delivery and severity of disease. There was no significant difference in the general data of the three groups of patients, and they were comparable (see Table 1).

**Table 1:** General information of patients

Index	Group A(*n*=30) (`x ± s)/ M(*P*_25_,*P*_75_)/ *n*(%)	Group B(*n*=30) (`x ± s)/ M(*P*_25_,*P*_75_)/ *n*(%)	Group C(*n*=30) (`x ± s)/ M(*P*_25_,*P*_75_)/ *n*(%)	F/Z/X²	*P*
Age(years)	40.47 ± 1.96	40.57 ± 1.83	39.93 ± 1.66	0.035	0.966
BMI(kg/m²)	24.32 ± 0.55	23.96 ± 0.65	24.55 ± 0.49	0.268	0.765
Disease duration (years)	3.00(1.75,7.00)	3.00(1.75,5.50)	3.00(1.00,5.00)	0.221	0.896
Production times				1.271	0.530
1	16(53.3)	18(60.0)	17(56.7)		
2	13(43.3)	9(30.0)	12(40.0)		
3	1(3.3)	3(10.0)	1(3.3)		
Mode of delivery				0.274	0.872
Vaginal delivery	27(90.0)	28(93.3)	27(90.0)		
Cesarean section	3(10.0)	2(6.7)	3(10.0)		
Degree of disease				0.211	0.900
Mild	17(56.7)	19(63.3)	18(60.0)		
Moderate	7(23.3)	5(16.7)	4(13.3)		
Severe	6(20.0)	6(20.0)	8(26.7)		

Note: BMI: body mass index; M:median

### Methods

#### Group A: Biofeedback Electrical Stimulation

The SUI treatment module of the PHENIX USB 4 pelvic floor rehabilitation therapy device was used. A pelvic floor muscle therapy head (vaginal electromyography type), connected to the PHENIX USB 4 pelvic floor treatment device, was gently inserted into the patient’s vagina. Special attention was paid to keeping the head’s metal electrode above the hymen margin, and patients were advised to remain still during the treatment process to avoid receiving burns. The electrical stimulation frequency was 50 Hz, the pulse width was 250 µs and the current intensity did not exceed 50 mA. The optimal intensity was when the patient could clearly feel the pelvic floor muscle twitching and could tolerate it without discomfort. The therapy was conducted three times a week for 5 weeks, with a duration of 30 min each time. Patients were then discharged with instructions to perform pelvic floor muscle training (PFMT), focusing on anal contractions. For this PFMT, the patient maintains normal breathing, relaxes the abdomen and legs and contracts the pelvic floor muscles to lift the anus.

#### Group B: Acupuncture

A single-use sterile needle of Changchun Aikang brand was selected, with a size of 0.30 × 40 mm. The Ding points were *Guanyuan* point, *Qihai* point, *Zhongji* point, *Zusanli* point, *Sanyinjiao* point and *Yinlingquan* point. Routine disinfection of the patient’s skin was performed, and the *Guanyuan*, *Qihai* and *Zhongji* points were punctured obliquely downward, with the needle inserted 1–1.2 cun, evenly lifted, inserted and twisted to obtain qi. At the same time, the moxa column was ignited and placed in the moxibustion box, with the box placed above the three points of *Guanyuan*, *Qihai* and *Zhongji* in the patient’s abdomen. The moxibustion was performed until the skin was red and the deep tissue was heated. The treatment was performed once a day, Monday–Friday, 30 min each time for a total of 10 times. The patients were instructed to return home for PFMT, with the same method used as that for group A.

#### Group C: Acupuncture Combined with Biofeedback Electrical Stimulation

Biofeedback electrical stimulation therapy combined with acupuncture and moxibustion was administered to the patients. After 10 sessions of acupuncture, the unfinished biofeedback electrical stimulation treatment was continued. The patients were instructed to return home for PFMT, with the same method used as that for groups A and B.

### Observation Indicators

#### Pelvic Floor Muscle Strength

The French Sugiyama PHENIX USB2 pelvic floor rehabilitation therapy instrument was used to detect the muscle strength of pelvic floor type I and type II muscle fibres. Class I muscle strength: amplitude height ≥ 40%, duration 0–5 s corresponds to 0–5 level muscle strength respectively; class II muscle strength: amplitude height ≥ 60%, duration 0–5 s corresponds to 0–5 level of muscle strength respectively.

#### One-Hour Urine Pad Test

The patient was asked to empty their bladder before the test, and the urine pad was weighed and placed in the perineum. At 0–15 min, the patient drank the mineral water prepared in advance. At 16–45 min, the patient walked as normal, going up and down the stairs. At 46–60 min, the patient ran on the spot for 1 min before sitting up continuously, coughing forcefully 10 times and bending over. The patient picked up 5 small objects on the ground before the patient was asked to wash their hands with tap water for 1 min. After 1 h, the patient was instructed to remove the urine pad and weigh it, retain the urine and measure the urine volume. A urine leakage of > 2 g is positive. Based on the amount of urine leakage, the degree of urinary incontinence can be divided as follows: Urinary leakage of 2–5 g (< 5 g) is considered mild, 5–10 g (< 10 g) is moderate, 10–50 g(<50 g) is severe, and over 50 g is extremely severe.

#### ICI-Q-SF Questionnaire

The patients reviewed their situation in the last 4 weeks and scored three questions: the frequency of urine leakage, the amount of urine leakage and the degree of impact on daily life. The scores were subsequently totalled; the higher the total score is, the more severe the symptoms of urinary incontinence and the greater the impact on life.

### Criteria for Assessing Efficacy

The effect was measured by the decrease in urine leakage in the 1-hour urine pad test before and after treatment ([initial urine leakage before treatment − urine leakage after treatment] ÷ initial urine leakage × 100%). A decrease of < − 25% indicates deterioration, a decrease of − 24–24% indicates no change, a decrease of 25–74% indicates slight improvement, a decrease of > 75% indicates clear improvement and a 100% decrease indicates cure. Total effective rate = ([number of cured cases + number of cases with obvious improvement + number of cases with slight improvement] ÷ total number of cases) × 100%.

### Statistical Analysis

The statistical analysis was performed using SPSS (ver. 26.0). Measurement data satisfying normal distribution were represented by ($\bar X \pm S$), with a t-test used for intra-group comparison and one-way analysis of variance used to analyse the differences between groups. The data that did not follow normal distribution (urine leakage volume in the 1-hour urine pad test and ICI-Q-SF questionnaire scores) were expressed as the median (interquartile range), with the Mann–Whitney U nonparametric test used to compare the differences before and after, and the Kruskal–Wallis nonparametric test used to analyse the differences between groups. The count data were expressed as percentages, and the chi-square (*χ*^*2*^) test was used for comparison between groups. The nonparametric rank-sum test was used for the rank data. A *P*-value of < 0.05 indicated that the difference was statistically significant. There was no difference between the non-transformed data and the transformed data.

## Results

### Comparison of General Data

There was no significant difference in age, body mass index, course of disease, number of deliveries, mode of delivery and disease severity among the three groups (all *P* > 0.05), which were comparable (see Table 1).

### Comparison of Pelvic Floor Muscle Strength

Before treatment, there was no significant difference in muscle strength of pelvic floor type I and type II muscles between the three groups (*P* > 0.05). After treatment, the muscle strength of type I and type II muscles in two groups (A and C) was significantly improved (*P* < 0.05), with the difference statistically significant. After treatment in group B, the muscle strength of type I and type II muscles was not significantly improved (*P* > 0.05), with the difference not statistically significant (see Tables 2 and 3).

**Table 2:** Muscle strength of class I muscle fibers [cases]

Group	Case	Grade before treatment							Grading after treatment						Z	*P*
		0	1	2	3	4	5		0	1	2	3	4	5		
A	30	14	6	0	4	2	4		0	1	5	7	4	13	4.311	<0.001
B	30	14	5	1	3	5	2		6	8	6	2	3	5	1.603	0.109
C	30	12	5	7	2	1	3		0	1	4	5	9	11	4.866	<0.001
*Z*		0.034							18.540							
*P*		0.983							<0.001^*^							

Note: *P < 0.05

**Table 3:** Muscle strength of class II muscle fibers [cases]

Group	Case	Grade before treatment							Grade after treatment						Z	*P*
		0	1	2	3	4	5		0	1	2	3	4	5		
A	30	14	7	1	2	1	5		4	2	3	6	3	12	3.354	0.001
B	30	15	4	4	2	3	2		9	7	5	3	3	3	1.230	0.219
C	30	11	4	3	5	1	6		0	3	5	5	5	12	3.256	0.001
Z		1.886							16.849							
*P*		0.390							<0.001^*^							

Note: *P < 0.05

### Comparison of 1-Hour Urine Pad Test Results

Before treatment, there was no significant difference in the urine leakage volume of the three groups of patients in the 1-hour urine pad test (*P* > 0.05). After treatment, the urine leakage improved more obviously in group C (see Table 4).

**Table 4:** 1 h urine pad test results [g, M (P25, P75)]

Group	Before therapy	After therapy	Z	*P*
A	3.60(2.68,6.98)	1.30(0.48,2.28)	-5.125	<0.001
B	2.75(2.30,6.93)	1.15(0.70, 2.28)	-4.698	<0.001
C	3.70(2.60,12.05)	0.50(0.10,1.50)	-6.133	<0.001
*Z*	1.555	7.882		
*P*	0.460	0.019		

### Comparison of ICI-Q-SF Questionnaire Scores

There was no significant difference in ICI-Q-SF questionnaire scores between the three groups before treatment (*P* > 0.05). After treatment, the questionnaire scores of the three groups were all decreased (*P* < 0.05), with the scores of group C decreasing more significantly than those of groups A and B (see Table 5).

**Table 5:** ICI-Q-SF questionnaire score [points, M (P25, P75)]

Group	Before therapy	After therapy	Z	*P*
A	9.00(5.00,11.25)	4.00(4.00,6.00)	-4.509	<0.001
B	9.00(5.75,11.25)	5.00(4.00,5.00)	-4.669	<0.0001
C	7.50(6.00,11.25)	4.00(0.00,4.00)	-5.893	<0.001
*Z*	0.263	9.592		
*P*	0.877	0.008*		

Note: *P < 0.05

### Treatment Effect

The therapeutic effects of the three groups were compared following treatment. The effect of group C was the most obvious (see Table 6).

**Table 6:** Analysis of treatment effect [cases (%)]

Group	No change	Slight improvement	Significant improvement	Cure	Efficient
A	4(13.3)	11(36.7)	13(43.3)	2(6.7)	26(86.7)
B	5(16.7)	13(43.3)	10(33.3)	2(6.7)	25(83.3)
C	1(3.3)	3(10.0)	20(66.7)	6(20.0)	29(96.7)
*Z*	14.148				
*P*	0.001				

## Discussion

In Western medicine theory, it is believed that SUI is due to pregnancy, childbirth, decreased oestrogen levels, chronic cough, long-term constipation, obesity and other reasons (Peng et al., 2020), resulting in decreased pelvic floor muscle strength, weak pelvic floor tissue, ligament and fascia laxity, and bladder neck. When the urethra moves down and the abdominal pressure increases, the urethral orifice cannot be closed properly. Therefore, improving pelvic floor muscle strength is the fundamental method to improve pelvic floor function. The current clinical methods to improve pelvic floor muscle strength are mainly PFMT and biofeedback electrical stimulation. The National Institute for Health and Care Excellence recommends professional-guided pelvic floor training for more than 3 months as the first-line treatment for SUI (level A evidence) (Thüroff et al., 2011). In the present study, all three groups of patients were supplemented with PFMT to exercise pelvic floor muscles and improve pelvic floor muscle strength. Although group B cooperated with the PFMT based on acupuncture, the pelvic floor muscle strength of the group B patients did not improve significantly. Studies have shown that PFMT has no significant effect on patients with severely low muscle strength (grade 0–1%) who cannot effectively actively contract the pelvic floor muscles (Hu et al., 2020). Prior to treatment, 53.3% of patients in group B had a muscle strength of grade 0–1%, which may have led to the lack of statistically significant improvement in muscle strength. In addition, the course of treatment was short, with only 10 days of PFMT potentially not effective. Some studies believe that the deep sacral nerves pass through the middle *liao* and sub *liao*, and deep puncturing of the two points can directly adjust the function of the lumbosacral autonomic nerve, increase the resistance of the urethra and increase the resistance of the pelvic floor muscles (Mo & Liu, 2013). The acupuncture points selected in this study did not significantly improve pelvic floor muscle strength.

However, in both groups A and C, biofeedback electrical stimulation technology was applied, and the muscle strength of these groups improved significantly. In this study, the patients were initially stimulated 1–3 times to arouse the proprioception of the patients and adjust the blood circulation, muscle sensitivity, nerve sensitivity and the number of muscles, especially for the patients with severely low muscle strength. The electrical stimulation through the stimulation nerves activate severely damaged pelvic floor cells, increase the number of muscle fibres and have a certain effect. The 4th–10th sessions with intermittent biofeedback electrical stimulation continued to improve pelvic floor muscle function and taught the patients to correctly contract and exercise pelvic floor type I and type II muscle fibres. For the 11th–15th sessions, a simple biofeedback mode was given to train patients to control muscle contractions in various situations without leakage of urine. Biofeedback electrical stimulation enables passive contraction and correct active contraction of pelvic floor muscles, improves pelvic floor muscle strength, and the treatment efficiency can reach 70–80% (Liu et al.,^2020^). A large number of studies have shown that biofeedback electrical stimulation is more effective than PFMT alone in the treatment of SUI (Aukee et al., 2004; Herderschee et al., 2013). Biofeedback electrical stimulation is particularly more intuitive and effective for patients who do not contract their pelvic floor muscles at first, and biofeedback electrical stimulation can help patients master muscle training skills and improve the accuracy and effectiveness of home-based PFMT.

The 1-hour urine pad test is an objective indicator for evaluating the amount of urine leakage of patients. In this study, the urine leakage of the three groups of patients was reduced in the urine pad test following treatment, and the reduction in group C was more obvious than that in groups A and B. The patients in group A used biofeedback electrical stimulation to improve pelvic floor function by enhancing pelvic floor muscle strength, so that the amount of urine leakage was reduced. Traditional Chinese medicine holds that ‘stress urinary incontinence’ belongs to the medical categories of ‘bladder cough’, ‘coughing and enuresis’, ‘drowning’ and ‘inability to urinate’. Although the disease location is in the bladder, the root is in the kidney. The kidney qi is deficient, the qi transformation is abnormal, the lower energy is depleted, the solid intake has no power and the bladder is missed. Acupuncture and moxibustion can warm the meridians, invigorate the kidney and strengthen the root, invigorate the spleen and replenish qi, warm the lower energy and stabilise the bladder, thereby alleviating the symptoms of SUI. There is a long history of research on the treatment of stress urinary incontinence through acupuncture, and it is the most recognised and accepted traditional Chinese medicine method in modern medicine (Chen et al., 2020). Patients in group B were treated with acupuncture and moxibustion. During the research process, it was found that acupuncture and moxibustion had a more rapid effect. Most patients began to relieve their urinary leakage symptoms after 3–4 treatments, and some patients experienced a decrease in urinary leakage after 1–2 acupuncture treatments.

In this study, the patients were treated 10 times in 2 weeks, and the symptoms of urinary leakage and quality of life were improved, with the short course of treatment an additional advantage of acupuncture treatment. At present, there are few stratified studies on the therapeutic effects of acupuncture and moxibustion on different degrees of SUI. In this experiment, a patient with severe SUI had a urine leakage of 29.4 g in the urine pad test 1 h before the test, but this decreased to 0.1 g following the full acupuncture treatment (i.e. cure). This suggests that acupuncture may have a more significant therapeutic effect on patients with severe SUI, while this requires further confirmation. Group C was treated with acupuncture combined with biofeedback electrical stimulation. Acupuncture can dredge the meridians, reconcile yin and yang, strengthen the body and eliminate pathogens, invigorate the kidney and strengthen the body, and absorb the body fluid and stop the waste. Biofeedback electrical stimulation can essentially activate muscle cells, improve the overall muscle strength of the pelvic floor, enhance urethral closure pressure and improve urinary continence. The combined application can not only significantly reduce the amount of urine leakage of patients, relieve their clinical symptoms and improve their quality of life but can also enhance the pelvic floor muscle strength, consolidate the curative effect and treat both internal and external, and the effect is enhanced.

Stress urinary incontinence is also known as ‘social cancer’ and seriously affects the patient’s life and interpersonal communication. The ICI-Q-SF questionnaire is a subjective index to evaluate the impact of urine leakage on daily life. The questionnaire includes the number of urine leakages, the amount of urine leakage and the impact on daily activities, such as housework and outdoor activities. In this study, the ICI-Q-SF questionnaire scores of the three groups of patients were decreased, with the decrease in group C more obvious than that in groups A and B.

A total of 90 patients were included in this study, and there were only 30 patients in each group. The sample size was not large, and there was no stratified study on the clinical efficacy of mild, moderate and severe patients; the patients’ efficacy remains unknown. We did not follow up on long-term effects after treatment. This is the work that needs to be conducted in future follow-up research.

## Conclusion

In conclusion, biofeedback electrical stimulation therapy can significantly improve the muscle strength of patients. Acupuncture and moxibustion therapy has a rapid effect and a short course of treatment. The combination of the two can effectively improve the symptoms of urinary leakage in female patients and improve pelvic floor muscle strength and pelvic floor function. The combined treatment is more effective in the improvement of the quality of life of patients compared with biofeedback or acupuncture alone, and clinical promotion can be attempted.

## Data Availability

All data generated or analyzed during this study are included in this published article.

## Declarations

### Clinical Trial Registered Number

ClinicalTrials.gov Identifier: NCT05531071.
